# Adaptive language control between comprehension and production in bilinguals

**DOI:** 10.1017/S1366728926101151

**Published:** 2026-03-25

**Authors:** Chuchu Li, Qi Cheng

**Affiliations:** 1 University of California San Diego, USA; 2 Linguistics, University of Washington, USA

**Keywords:** bilingual language control, cross-modality language switching, language cues, within-modality control demands, adaptive control

## Abstract

This study investigates bilingual language control across comprehension and production. In three experiments, Chinese–English bilinguals alternated between tasks on every trial. In the comprehension task, participants judged the meaning of a written word (Experiment 1) or a spoken word (Experiments 2 and 3) in either language. In the production task, they named pictures in only one language (Experiments 1 and 2) or in either language (Experiment 3), with half of the trials involving language switching. Thus, salient visual language cues were available only in Experiment 1, and within-production language switching occurred only in Experiment 3. Language switch costs from comprehension to production emerged only in Experiment 2, where spoken word comprehension was paired with single-language production. These findings suggest that reduced saliency of language cues encourages a shared language control mechanism across comprehension and production when within-production control demands are low, supporting the adaptive nature of bilingual language control.

## Highlights

- Bilingual language control between comprehension and production is adaptive.
- Salient language cues help reduce cross-modality language switch costs.
- Within-modality language control demands override those across modalities.

## 1. Introduction

It is common to observe bilinguals switch languages when communicating with other bilinguals. They rarely produce the unintended language by accident, showing remarkable language control abilities. Prominent models of bilingual language production suggest that this control is achieved through inhibiting the nontarget language. According to the Inhibitory Control Model (ICM; Green, 1998), both languages of bilinguals are activated when only one is produced, and bilinguals inhibit the nontarget language in proportion to its activation level. When this inhibited language needs to be produced again (i.e., language switching), bilinguals must overcome previously applied inhibition, resulting in switch costs (e.g., Meuter & Allport, 1999). Such switch costs were typically larger for the dominant language than for the nondominant language, as overcoming stronger inhibition requires more time (see Bobb & Wodniecka, 2013; Declerck & Koch, 2023; Declerck & Philipp, 2015, for a review).

In comprehension, language control appears to rely on different mechanisms (Ahn et al., 2020; Mosca & de Bot, 2017). Activation-based frameworks such as the Bilingual Interactive Activation Plus model (BIA+; Dijkstra & van Heuven, 2002), the *Multilink* model (Dijkstra et al., 2019) and the Bilingual Language Interaction Network for Comprehension of Speech model (*BLINCS*; Shook & Marian, 2013) proposed that comprehension is achieved through graded activation and cross-linguistic integration rather than through inhibition of the nontarget language. Supporting this view, reading silently (comprehension) versus reading aloud (involving production) elicited different switch cost patterns even for identical materials: the former tended to elicit symmetrical switch costs, whereas the latter produced higher costs on the dominant language (Li et al., 2024). The symmetry in comprehension likely arises because control is achieved by adjusting task schemas to bias activation toward the target language, a process that imposes comparable costs across both language directions. Interestingly, however, some studies suggested that comprehending one language can elicit inhibition on bilinguals’ other language in production, leading to language switch costs from comprehension to production (thus shared language control mechanisms between comprehension and production) when bilinguals alternate between these two language tasks, a common situation in real life, such as during conversations (Gambi & Hartsuiker, 2016; Li & Gollan, 2022; Peeters et al., 2014). These findings align with the BIA-d model (Grainger et al., 2010), which posits that the activation of lexical items in the target language and inhibition of lexical items in the nontarget language are modulated by language nodes, which can be affected by both exogenous and endogenous factors (e.g., comprehending a word versus the intention to speak in one language).

### 1.1. Adaptive language control within and across modalities

Language control is thought to be adaptive. According to the widely accepted Adaptive Control Hypothesis (ACH), bilinguals flexibly regulate control processes to match the unique demands of their interactional context (Green & Abutalebi, 2013; Green & Wei, 2014). For instance, in a single- or dual-language context, the language task schemas are in a competitive relationship, and bilinguals may rely heavily on inhibition of the nontarget language. In dense code-switching contexts, however, inhibitory control is more relaxed, and the language task schemas are in a cooperative relationship to allow for opportunistic planning and rapid language alternation. Supportively, Olson (2016) showed asymmetrical switch costs in a predominantly English or Spanish context (like a single-language context) but symmetrical costs in a balanced bilingual context in picture naming, suggesting that the degree of inhibition is modulated by the broader language context. This adaptivity may extend beyond linguistic contexts to include the modality of the language task itself. For example, bilinguals may adaptively adopt different control mechanisms based on the processing route, like whether they transition from comprehension to production or across different output channels (i.e., within production). Such cross-modality transitions may require the system to adaptively calibrate how much inhibition is necessary to suppress a recently processed word in one modality (e.g., a word in comprehension) to allow for successful production in another.

Indeed, Li and Gollan (2022) suggested that language control is adaptive across modalities through the comparison between two experiments. In their Experiment 2, when alternating between semantic judgment on visual words and picture naming, Spanish–English bilinguals’ picture-naming latencies in the dominant language were slower when pictures were preceded by a visual word in the nondominant language than by a word in the dominant language. Such switch costs were not shown in the nondominant language, suggesting that language control across comprehension and production was exerted mainly on the dominant language. Similar results were observed by Peeters et al. (2014) in French–English bilinguals, who alternated between semantic judgment or language membership judgment and picture naming. Together, these studies showed that comprehension can trigger control adjustments affecting subsequent production. Crucially, because pictures in these studies were named in only one language per block, the possibility of switching within the production system was eliminated. This confirms that the observed control adjustments were driven entirely by the preceding comprehension task, demonstrating a direct cross-modality influence. Despite this clear evidence that comprehension can trigger inhibitory control that spills over to the following production trial, the nature of this mechanism may change depending on specific task demands. Experiment 1 in Li and Gollan (2022) showed that, when alternating between reading aloud words and naming pictures, the language of the preceding visual word did not affect picture naming in either language. This contrast suggests that bilinguals do not use a “one-size-fits-all” control system when alternating between comprehension and production. Instead, they adaptively employ different mechanisms depending on the specific task. Following this, the present study aimed to further investigate in detail what factors modulate this adaptive cross-modality language control.

### 1.2. The role of language cues

Li and Gollan (2022) proposed that bilingual language control between comprehension and production depends on the effectiveness of language cues. Reading aloud and picture naming share the same intention – to name the stimuli, thus leading to low task demands, so that bilinguals can effectively utilize visual cues (words versus pictures) to guide language selection in both tasks. In contrast, when alternating between semantic judgment and picture naming or between passive listening and picture naming (as in Gambi & Hartsuiker, 2016), the intention was different in comprehension and production tasks (not naming versus naming). In addition, semantic judgment was less naturalistic than word naming, resulting in high task demands. With limited cognitive resources, bilinguals were unable to use visual cues (pictures versus words) effectively in language selection and thus strongly inhibited their dominant language throughout the entire testing block. Nevertheless, the effectiveness of language cues was not directly manipulated in that study, a gap addressed in the present study.

In some other studies, however, direct manipulation suggested that language cues modulate inhibitory control within production. In particular, some inherent features of stimuli associated with language (or natural language cues) can largely facilitate language selection, reducing or even eliminating language switch costs (Li et al., 2013; Liu et al., 2019; Martin et al., 2016; Molnar et al., 2015; Woumans et al., 2015). For example, when Arabic–English bilinguals were cued to switch language in picture naming according to color patches (e.g., a green or a red square, an arbitrary language cue), they showed significant language switch costs; however, when being cued according to interlocutors’ identity (e.g., an interlocutor who can only speak Arabic or English, a nonarbitrary language cue), no significant language switch costs were shown (Blanco-Elorrieta & Pylkkänen, 2017). Orthographic information of distinct scripts – another inherent language feature – plays a similar role. In Fadlon et al. (2019), Spanish–English and Hebrew–English bilinguals read aloud paragraphs mixed with their two languages. Hebrew–English bilinguals with distinct scripts produced fewer cross-language intrusion errors than Spanish–English bilinguals, revealing significantly smaller language switch costs. Salient orthographic cues reduce the demands of inhibitory control, probably because they make visual words in two languages immediately and reliably distinguishable, even before lexical access (Hoversten et al., 2017).

If language cues also modulate inhibitory control between comprehension and production, when alternating between picture naming in a single language and visual word recognition in either language, orthographic information in distinct scripts may largely facilitate language selection in all trials,^1^ reducing the need to strongly inhibit the dominant language. As a result, bilinguals with languages in distinct scripts (e.g., Chinese–English bilinguals) may not show language switch costs from reading comprehension to production. However, such benefits might be absent when these bilinguals alternate between listening comprehension and production, as phonological and phonetic cues are typically less immediately available and less perceptually salient than orthographic cues (Carrasco-Ortiz & Frenck-Mestre, 2014).

### 1.3. The demands of language control within production

Another factor that may modulate language control between comprehension and production is language control demands within production. This claim is based on the findings of two recent studies that did not find switch costs from comprehension to production (Liu et al., 2020, 2021). In these studies, Chinese–English bilinguals alternated between listening comprehension and production in the same block, with both languages used in both tasks. In addition, they may name pictures in two or more consecutive trials, allowing for assessing within-production language switch costs. Participants never showed language switch costs from comprehension to production, but demonstrated robust switch costs within production. Liu et al. suggested that language competition within production may overshadow comprehension-based influences. When language control within production is challenging, the primary goal is to manage cross-language interference from the internal language system, rendering the influence of exogenous factors minimal. In contrast, when production is limited to one single language (e.g., Gambi & Hartsuiker, 2016; Li & Gollan, 2022; Peeters et al., 2014), the secondary source of cross-language interference (i.e., from exogenous factors) becomes salient and starts to play a role.

However, Liu et al. did not conduct an experiment with only one language allowed in production for direct comparison, leaving their hypothesis unconfirmed. In addition, involving both languages in production does not guarantee cost-free language switching from comprehension to production. In Li and Gollan (2021), Spanish–English bilinguals read aloud single-language sentences presented word by word. In each sentence, one word was replaced by a picture with a language cue on the top (e.g., I want to print a picture of a [tooth] and use it in the next class; “tooth” was replaced by a picture with a US flag or a Mexican flag on the top), and participants named the picture in the cued language. Despite production involving both languages, naming the picture in a language different from the sentence context produced switch costs. Given that reading aloud in one language did not elicit language switch costs in the following single-language picture-naming trial in Li and Gollan (2022), production involving both languages may even encourage language switch costs across language tasks/modalities due to enhanced control demands, challenging Liu et al.’s hypothesis.

### 1.4. The present study

The present study investigated bilingual language control across comprehension and production in different contexts, focusing on how comprehension affects language control in production. We examined two key factors: (a) the effectiveness of language cues and (b) the demands of language control within production. For (a), we investigated language switching from reading comprehension to production (Experiment 1) and from listening comprehension to production (Experiment 2) among Chinese–English bilinguals, with only the former involving distinct orthographic information to facilitate language selection. For (b), we compared conditions where only one language was used in production (Experiment 2) versus both languages (Experiment 3), with the latter context eliciting stronger language competition within production than the former.

## 2. Experiment 1: Reading comprehension and single-language picture naming

First, we examined whether results in Peeters et al. (2014) and in Li and Gollan (2022); Experiment 2) can be replicated in different-script bilinguals, with only one change in the design: while most of the comprehension trials in these two studies elicited the “no” response, we had equal number of “yes” and “no” responses. This reduced participants’ tendency to provide a “no” response quickly without processing the lexical information of a word, improving the validity of the comprehension trials. Following these two studies, Chinese–English bilinguals alternated between semantic judgment of a visual word and picture naming on every trial, with picture naming always in one single language in each block. If different-script bilinguals are able to use language cues to guide language selection effectively throughout a testing block, we may observe minimal language switch costs from comprehension to production in Experiment 1.

### 2.1. Method

#### 2.1.1. Participants

Thirty-two^2^ Chinese–English bilingual undergraduates at the University of California, San Diego, participated for course credit (9 males and 23 females); each participant signed an IRB-approved informed consent form. One participant was removed from data analysis due to a low accuracy rate in the comprehension trials (<80%). Table 1 shows remaining participants’ characteristics and *Multilingual Naming Test* scores in both languages, which were used to measure bilinguals’ language proficiency (MINT Sprint 2.0; Garcia & Gollan, 2022; Gollan et al., 2024; Neveu et al., 2025). According to this measurement, 25 participants were Chinese-dominant and six were English-dominant. According to the ICM, language switch costs were more about language dominance rather than language per se, so we presented data by language dominance (dominant versus nondominant) instead of by language (English versus Chinese), also following Li and Gollan (2022).

**Table 1.** Means and standard deviations of participant characteristics

Characteristic	Experiment 1		Experiment 2		Experiment 3		Experiments 1 vs. 2 *p* value	Experiments 2 vs. 3 *p* value
	*M*	*SD*	*M*	*SD*	*M*	*SD*		
Age	20.9	2.3	20.4	1.1	21.7	1.90	0.35	0.09
Age of acquisition of Chinese	0	0	0	0	0	0	/	/
Age of acquisition of English	4.3	3.0	5.8	3.0	5.5	3.1	0.13	0.61
Self-rated Chinese speaking proficiency^a^	6.7	0.6	6.6	0.9	6.9	0.4	0.74	0.15
Self-rated English speaking proficiency^a^	5.6	1.0	5.6	0.9	5.7	1.1	0.96	0.61
Self-rated Chinese understanding proficiency^a^ ^,^^b^	6.7	1.0	6.8	0.5	6.9	0.4	0.71	0.22
Self-rated English understanding proficiency^a^ ^,^^b^	5.4	1.0	5.8	0.9	5.8	0.9	0.20	0.61
Current percent of English use	57.9	23.2	53.0	17.6	59.3	24.2	0.37	0.31
Percent of English use during childhood	33.0	18.2	36.0	21.9	33.2	22.4	0.56	0.57
MINT Sprint 2.0 score in Chinese^c^	64.7	6.5	62.6	10.9	63.0	10.8	0.39	0.58
MINT Sprint 2.0 score in English^c^	51.8	13.5	51.2	11.0	49.5	12.9	0.85	0.49
MINT Sprint 2.0 score in the dominant language^c^	67.3	4.7	67.4	3.9	67.4	6.6	0.88	0.97
MINT Sprint 2.0 score in the nondominant language^c^	49.2	10.9	46.4	8.3	45.1	8.8	0.28	0.75

*Note:* Participants across different experiments were NOT significantly different from each other on any measurement, including MINT Sprint 2.0 scores of both languages, which were used to calculate Bilingual Index Scores.

a Proficiency-level self-ratings were obtained using a scale from 1 (almost none) to 7 (like a native speaker).

b Understanding refers to reading comprehension in Experiment 1 versus listening comprehension in Experiments 2 and 3, given that these are the relevant skills in the comprehension tasks in corresponding experiments.

c The maximum possible MINT Sprint 2.0 score is 80.

#### 2.1.2. Design and materials

Twenty-five line drawings were selected from the CRL International Picture Naming Project, each depicting a concept with a high-frequency name in both Chinese and English (Bates et al., 2003). None of these pictures had names that were cognates or cross-language homographs between the two languages, and none depicted animals. In addition, 50 English words and their Chinese translation equivalents – none of which overlapped with the picture names – were selected (see Appendix). These included 25 animal words and 25 nonanimal words. Within each language, word frequency was matched between the animal and nonanimal categories (*ps >* .12). Word frequency information was acquired from the SUBTLEX-US database for English (Brysbaert & New, 2009) and SUBTLEX-CH database for Chinese (Cai & Brysbaert, 2010).

Each participant completed two testing blocks, each consisting of 100 trial sets. In each trial set, a visual word was presented, followed by a picture. Specifically, the 25 pictures repeated four times within a block, once preceded by a Chinese animal word, once by an English animal word, once by a Chinese nonanimal word, and once by an English nonanimal word. Participants decided whether each visual word referred to an animal by pressing a “yes” or “no” button and named all pictures in one language per block, with the order of naming languages counterbalanced across participants. To minimize priming effects, visual words and their subsequent pictures were never semantically or phonologically related, and repeated presentations of the same picture were spaced at least five trials apart.

#### 2.1.3. Procedure

Pictures and words were presented using the DMDX software (Forster & Forster, 2003) on a computer with a 24-in. screen. Following Peeters et al. (2014), in each block, each trial set began with a fixation point (+) for 200 ms, followed by a blank (100 ms), a word (1500 ms), a blank (500 ms) and finally a picture (4000 ms). In other words, participants alternated between comprehension and production on every trial, so that each trial was a task switch trial, but only half were language switch trials. Bilinguals were instructed to make semantic judgments on words and name pictures as quickly and accurately as possible. Stimuli disappeared after a button press or when a spoken response was registered by the voice-key. Before each block, participants were first pre-exposed to all pictures to get familiar with their target names. Next, participants completed 10 practice trials with interwoven words and pictures that would not appear in the formal testing block. The training and practice sessions were repeated after the first testing block, but with names in the language to be used to name pictures in the second block.

### 2.2. Results

Analyses were carried out in R (R Core Team, 2013) with the lme4 package (Bates et al., 2015) for linear mixed-effects modeling (LMM) and general linear mixed-effects modeling (GLMM). Response time (RT) data for incorrect responses were excluded for both picture naming (1.0%) and semantic judgment trials (2.4%). Correct picture-naming RTs were trimmed if any one or more of the following conditions were met: stuttering, the correct answer failed to trigger the voice key, an error was made on the previous comprehension trial,^3^ or participants completed an incorrect task (e.g., reading a word aloud or categorizing a picture). Lastly, responses faster than 200 ms were removed (as were any responses above 4000 ms; these were not recorded), and responses above 3 standard deviations from the mean (per participant) were also removed. These trimming procedures excluded another 4.8% of picture-naming trials and 1.9% of semantic judgment trials.

#### 2.2.1. Picture naming

Following Li and Gollan (2022), contrast-coded fixed effects included language (dominant versus nondominant), trial type (switch versus nonswitch), block order (picture naming in the dominant language first versus nondominant first) and all the two-way and three-way interactions.^4^ Subjects and items were entered as two random intercepts with all related random slopes (correlation between random effects was dropped to avoid the failure of convergence). The same fixed and random effects were included in the logistic regression for analyses of error rates. The significance of fixed effects was assessed via likelihood ratio tests (Barr et al., 2013).

**Figure 1.**
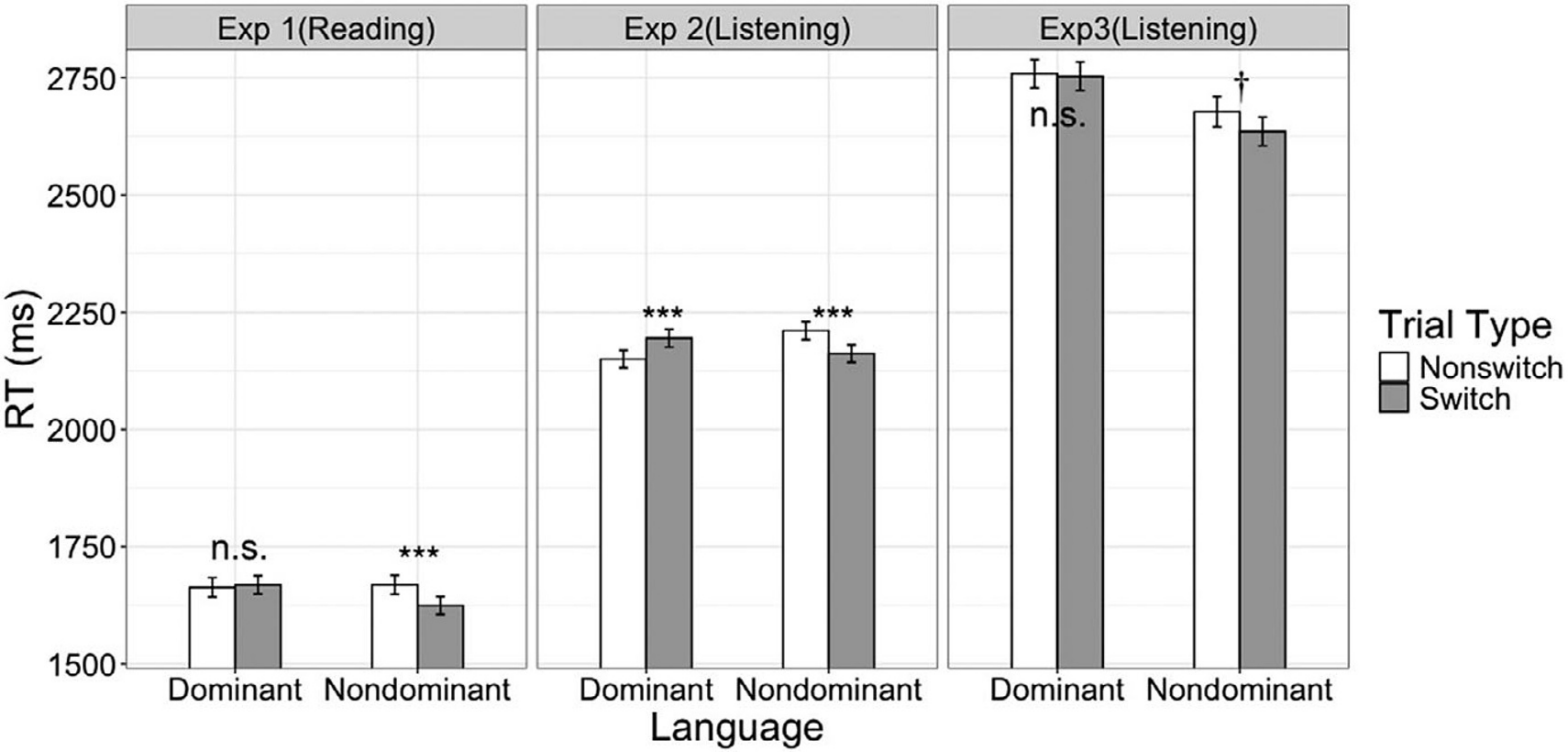
Mean *picture-naming* response time for each trial type and language in Experiment 1 (reading comprehension + single-language picture naming), Experiment 2 (listening comprehension + single-language picture naming) and Experiment 3 (listening comprehension + mixed-language picture naming). Error bars represent 95% confidence intervals. The most critical comparison was between switch and nonswitch bars in the dominant language, which showed switch costs only in Experiment 2 but no clear difference in Experiments 1 and 3. All three experiments showed switch benefits (or clear trend of switch benefits) in the nondominant language (n.s.: nonsignificant; ^†^*p* < .10; ***p < .001).

Figure 1 (left panel) shows the mean RTs per language and per trial type. Bilinguals named pictures faster on switch than nonswitch trials (*M* = 1647 ms versus 1666 ms; *β* = −19 ms, 95% CI = [−35 ms, −4 ms], *χ* ^2^ (1) = 5.78, *p =* .016), but this was qualified by a significant language * trial type interaction (*β* = 51 ms, 95% CI = [7 ms, 96 ms], *χ* ^2^ (1) = 4.93, *p =* .026). In the dominant language, participants responded to switch and nonswitch trials equally fast (*M =* 1669 ms versus 1663 ms; *p =* .67). The block order main effect and the block order * trial type interaction were not significant either (*ps >* .51). In the nondominant language, bilinguals responded faster to switch trials (*M =* 1624 ms versus 1669 ms; *β* = −45 ms, 95% CI = [−70 ms, −20 ms], *χ* ^2^ (1) = 12.08, *p* < .001). The block order * trial type interaction was not significant (*p =* .14), but the overall picture-naming speed in the nondominant language was faster in the second block, likely a practice effect (*M =* 1548 ms versus 1710 ms; *β* = −193 ms, 95% CI = [−367 ms, −19 ms], *χ* ^2^ (1) = 4.70, *p =* .030). In summary, in Experiment 1, bilinguals did not show switch costs in any situation.

Bilinguals named pictures in the dominant language more slowly than in the nondominant language if they completed the dominant language block first (*M =* 1699 ms versus 1548 ms), while they named pictures faster in the dominant language if they completed that block last (*M =* 1646 ms versus 1710 ms), a significant language * block order interaction that may reflect a practice effect (*β* = 135 ms, 95% CI = [37 ms, 233 ms], *χ* ^2^ (1) = 7.40, *p =* .006). Overall, there was no significant difference between the dominant and the nondominant languages (*M* = 1666 ms versus 1647 ms; 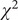 (1) < 1, *p* = .55). Table 2 shows the mean error rates for different languages and trial types. The analysis of error rates did not show any significant results (*ps >* .22).

**Table 2.** Mean *picture-naming* error rates (%; 95% confidence intervals in brackets) in Experiment 1 (reading comprehension + single-language picture naming), Experiment 2 (listening comprehension + single-language picture naming) and Experiment 3 (listening comprehension + mixed-language picture naming)

Language	Trial type	Experiment 1 (reading)	Experiment 2 (listening)	Experiment 3 (listening)
Dominant	Nonswitch	1.16 [0.63, 1.69]	0.64 [0.22, 1.06]	4.93 [3.84, 6.03]
	Switch	1.1 [0.58, 1.62]	0.5 [0.13, 0.87]	3.8 [2.83, 4.76]
Nondominant	Nonswitch	0.77 [0.33, 1.21]	0.86 [0.38, 1.34]	2.47 [1.68, 3.25]
	Switch	1.03 [0.53, 1.53]	0.5 [0.13, 0.87]	1.33 [0.75, 1.91]

*Note:* The error rates were overall low (<5%), without significant switch effects in any language or any experiment.

#### 2.2.2. Semantic judgment

Table 3 shows RTs and error rates of semantic judgment responses in all conditions. The method of analyzing semantic judgment data followed that of picture-naming trials. However, in these analyses, picture-naming language determined the trial type of words – in each block, all words in the same language as the picture-naming trials were nonswitch trials, while all other words were switch trials. Put differently, switch and nonswitch trials for the same language occurred in different blocks. Therefore, it is difficult to differentiate switch effects from language or order effects for word responses, limiting the interpretive value of these data. Therefore, following Peeters et al. (2014) and Li and Gollan (2022), we briefly reported the semantic judgment results but focused our discussion exclusively on the picture-naming results.

**Table 3.** Mean response times and error rates (95% confidence intervals in brackets) for *semantic judgment* in Experiment 1 (reading comprehension + single-language picture naming)

Word language	Picture-naming block order	Word trial type	RT (ms)	Error rate (%)
Dominant language	Dominant first	Nonswitch (i.e., in Block 1)	753 [736, 770]	2.64 [1.32, 3.95]
		Switch (i.e., in Block 2)	666 [651, 681]	2.11 [0.93, 3.29]
	Nondominant first	Nonswitch (i.e., in Block 2)	717 [701, 734]	2.21 [1.25, 3.16]
		Switch (i.e., in Block 1)	771 [754, 788]	1.77 [0.91, 2.62]
Nondominant language	Dominant first	Nonswitch (i.e., in Block 2)	694 [680, 709]	2.06 [0.90, 3.21]
		Switch (i.e., in Block 1)	770 [754, 785]	2.57 [1.29, 3.86]
	Nondominant first	Nonswitch (i.e., in Block 1)	829 [812, 847]	2.51 [1.49, 3.52]
		Switch (i.e., in Block 2)	734 [718, 751]	2.51 [1.49, 3.52]

*Note:* For the dominant language, switching elicited costs in RTs only if the nondominant language block was completed first, and elicited benefits in RTs if the dominant block was completed first; the same pattern was observed for the nondominant language, suggesting an order effect.

For RTs, bilinguals overall responded to words in the dominant language faster than in the nondominant language, a typical language dominance effect (*M =* 731 ms versus 762 ms; *β* = −34 ms, 95% CI = [−50 ms, −19 ms], 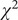 (1) = 17.37, *p* < .001). There was also a significant trial type main effect, showing switch benefits (*M =* 731 ms versus 762 ms; *β* = −16 ms, 95% CI = [−26 ms, −5 ms], 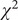 (1) = 8.28, *p =* .004). However, these effects were qualified by a significant three-way interaction (*β* = −243 ms, 95% CI = [−343 ms, −143 ms], 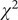 (1) = 22.69, *p* < .001). For both the dominant and nondominant languages, bilinguals showed switch benefits when nonswitch trials of this language were in the first block (*M difference =* −87 ms versus −95 ms, respectively), and showed switch costs when switch trials of this language were in the first block (*M difference =* 54 ms versus 76 ms, respectively) (see Table 3 for details). In other words, whichever trial type was completed in the second block was judged faster, likely a practice effect that was unsurprisingly stronger in the nondominant language. The analysis of error rates did not reveal any significant results (*ps >* .05).

### 2.3. Discussion

Inconsistent with Peeters et al. (2014) and Li and Gollan (2022), semantic judgment of visual words did not elicit language switch costs in either language on subsequent picture-naming trials, regardless of the block order. Additionally, bilinguals’ picture-naming responses were faster in whichever language was used in the second block, likely a practice effect. Also, we replicated switch benefits when bilinguals named pictures in their nondominant language. Critically, the absence of language switch costs from comprehension to production was consistent with our hypothesis that orthographic difference might serve as an effective language cue throughout the experiment, so that participants could rely on it to complete language selection easily in every single trial, avoiding strong inhibition on the dominant language throughout the whole testing block. Alternatively, the absence of language switch costs from comprehension to production could be due to the fact that Chinese and English are overall more typologically distinct than similar-script language pairs. If so, the same results should be observed when Chinese–English bilinguals alternate between listening comprehension and production.

## 3. Experiment 2: Listening comprehension and single-language picture naming

Experiment 2 aimed to test language switch effects when Chinese–English bilinguals alternate from listening comprehension to production, in which salient orthographic language cues are unavailable.

### 3.1. Method

#### 3.1.1. Participants

Thirty-two unbalanced Chinese–English bilingual undergraduates who did not participate in Experiment 1 were recruited from the same subject pool and participated for course credit (6 males and 26 females). Two subjects were removed due to a low accuracy rate in comprehension (<80%), and two were removed because of too many invalid production trials (>20% were named incorrectly, triggered by noise, or no response within 4000 ms). Table 1 shows self-reported participant characteristics and MINT Sprint 2.0 scores in English and Chinese of the remaining participants. Twenty-three remaining participants were Chinese-dominant, and five were English-dominant. Participants from Experiments 1 and 2 did not differ in either self-reported or objectively measured proficiency levels of the two languages or any other variables (*ps >* .10; see Table 1).

#### 3.1.2. Design, materials and procedure

The design, materials and procedures were the same as those in Experiment 1, except that all comprehension trials were auditory words. Auditory words were recorded by an early Chinese–English bilingual at a normal speed in a soundproof room (production duration of each word < 1500 ms). If participants pressed the button before the end of an auditory word, the sound stopped immediately.

### 3.2. Results

The procedures of data trimming and analyses were identical to those in Experiment 1.

#### 3.2.1. Picture naming

For RTs, we removed 0.6% incorrect responses and another 4.0% of trials due to trimming procedures. Figure 1 (middle panel) shows the mean RTs per language and per trial type. Overall, participants responded to switch and nonswitch trials equally fast (*M =* 2178 ms versus 2180 ms; *β* = −4 ms, 95% CI = [−19 ms, 11 ms], 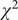 (1) < 1, *p =* .61), but all two-way and three-way interactions were significant (*p*s < .05). Thus, following Experiment 1, we further conducted analyses on each language separately.

**Figure 2.**
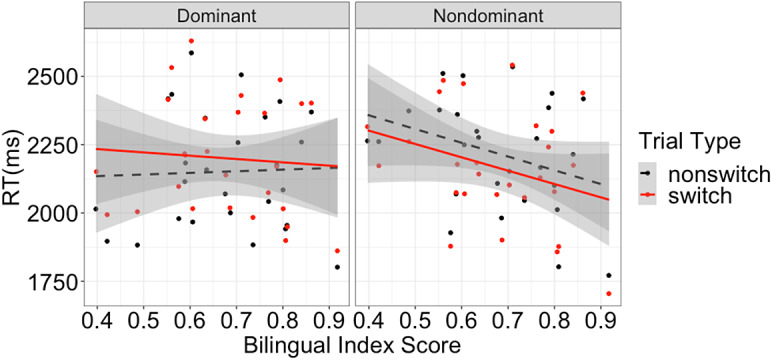
Mean *picture-naming* response time by trial type and language as a function of Bilingual Index Score (BIS) in Experiment 2 (*n* = 28; more balanced bilinguals have higher Index Scores). Each panel included nonswitch and switch data points of each participant in the corresponding language. The difference between the switch and nonswitch lines suggested switch effects (costs in the dominant language, left panel, versus benefits in the nondominant language, right panel). Critically, with increased BIS, costs become smaller while benefits remain the same.

For the dominant language, participants responded to switch trials more slowly than to nonswitch trials, showing significant language switch costs (*M =* 2194 ms versus 2150 ms, *β* = 44 ms, 95% CI = [19 ms, 68 ms], 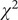 (1) = 11.87, *p* < .001). The block order main effect and the block order * trial type interaction were not significant (*ps >* .17). Following Li and Gollan (2022), we additionally explored whether switch costs in the dominant language were modulated by bilinguals’ degree of bilingualism, which was quantified by centered Bilingual Index Score (BIS). In this analysis, fixed effects included trial type, block order, centered BIS and all two-way and three-way interactions. We calculated BIS by dividing MINT Sprint 2.0 scores in the nondominant language by the dominant language, such that the BIS was a continuous variable and a BIS closer to 1 would indicate more balanced bilinguals. As shown in Figure 2 (left panel), switch costs were stronger among more unbalanced bilinguals, a significant interaction between BIS and trial type ( 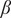 = − 208 ms; 95% CI = [−381 ms, −36 ms], 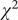 (1) = 5.51, *p =* .019).^5^ BIS did not show significant main effects or interactions with any other factors (*ps >* .10).

For the nondominant language, bilinguals responded faster to switch trials than to nonswitch trials, replicating switch benefits in Experiment 1 (*M =* 2162 ms versus 2211 ms, 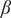 = − 51 ms; 95% CI = [−73 ms, −30 ms], 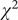 (1) = 21.51, *p* < .001). However, this time switch benefits were exclusively driven by participants who completed the nondominant language block first (*M difference =* 102 ms versus −5 ms for nondominant first versus dominant first, respectively; 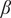 = 102 ms; 95% CI = [59 ms, 145 ms], 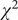 (1) = 21.29, *p* < .001). In other words, in the second block, in which participants became more used to the paradigm, switch benefits disappeared. Additional analysis with centered BIS did not show a significant BIS * trial type interaction ( 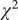 (1) < 1, *p =* .75; see Figure 2, right panel) or any other BIS effects (*ps >* .05).

Most critically, in Experiment 2 bilinguals showed switch costs on the dominant language regardless of block order, and this effect was stronger among unbalanced bilinguals; for the nondominant language, significant switch benefits were replicated but only among those who completed the nondominant language block first (i.e., when participants were not familiar with the paradigm).

Like Experiment 1, in Experiment 2 participants also showed a significant language * block order interaction ( 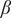 = 110 ms; 95% CI = [18 ms, 202 ms], 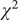 (1) = 5.51, *p =* .019). Again, bilinguals named pictures in the dominant language more slowly than in the nondominant language if they completed the dominant language block first (*M =* 2228 ms versus 2185 ms), while they named pictures faster in the dominant language if they completed that block last (*M =* 2116 ms versus 2186 ms), likely a practice effect. Table 2 shows the mean error rates for different languages and trial types, the analysis of which did not show any significant results (*ps >* .10).

The most critical contrast between Experiments 1 and 2 was that language switch costs from comprehension to production on the dominant language were only significant in Experiment 2, but not in Experiment 1. Therefore, we conducted a cross-experiment comparison focusing on the dominant language, with experiment, trial type, block order and all interactions as fixed effects. Subjects and items were entered as two random intercepts with related main effects as random slopes (trial type for subjects; experiment, block order and trial type for items; interaction terms were removed due to the failure to converge). This analysis revealed a significant interaction between experiment and trial type ( 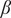 = 38 ms; 95% CI = [3 ms, 72 ms], 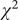 (1) = 4.60, *p =* .032), supporting significant cross-experiment difference on language switch costs.

#### 3.2.2. Semantic judgment

Table 4 shows RTs and error rates for semantic judgment responses in all conditions. In the RT analysis, we removed 1.7% incorrect responses and another 1.7% of trials due to trimming procedures. RT results were consistent with those in Experiment 1, with longer RTs in Experiment 2 than in Experiment 1 (*M =* 1296 ms versus 747 ms; 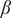 = 547 ms; 95% CI = [477 ms, 616 ms], 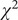 (1) = 238.12, *p* < .001). Bilinguals overall responded to words in the dominant language faster than in the nondominant language, a typical language dominance effect (*M =* 1271 ms versus 1320 ms; 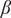 = − 64 ms; 95% CI = [−84 ms, −43 ms], 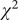 (1) = 36.54, *p* < .001). For both dominant and nondominant languages, bilinguals showed switch benefits when nonswitch trials of this language were in the first block (*M difference =* −19 ms versus −133 ms) and showed switch costs when switch trials of this language were in the first block (*M difference =* 58 ms versus 59 ms) (see Table 4 for details). That is, a word was always responded faster when appearing in the second block, particularly for the nondominant language, suggesting that the complicated block order * language * trial type interaction should be a practice effect ( 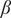 = − 266 ms; 95% CI = [−374 ms, −158 ms], 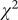 (1) = 23.19, *p* < .001).

**Table 4.** Mean response times and error rates (95% confidence intervals in brackets) for *semantic judgment* in Experiment 2 (listening comprehension + single-language picture naming)

Word language	Picture-naming block order	Word trial type	RT (ms)	Error rate (%)
Dominant language	Dominant first	Nonswitch (i.e., in Block 1)	1307 [1287, 1328]	0.90 [0.18, 1.62]
		Switch (i.e., in Block 2)	1288 [1266, 1310]	1.20 [0.37, 2.04]
	Nondominant first	Nonswitch (i.e., in Block 2)	1216 [1196, 1235]	1.20 [0.37, 2.03]
		Switch (i.e., in Block 1)	1274 [1257, 1291]	0.45 [−0.06, 0.96]
Nondominant language	Dominant first	Nonswitch (i.e., in Block 2)	1302 [1282, 1322]	2.65 [1.44, 3.85]
		Switch (i.e., in Block 1)	1361 [1340, 1382]	3.24 [1.91, 4.57]
	Nondominant first	Nonswitch (i.e., in Block 1)	1375 [1356, 1394]	2.06 [0.99, 3.13]
		Switch (i.e., in Block 2)	1242 [1224, 1261]	2.21 [1.1, 3.32]

*Note:* Consistent with Experiment 1, for the dominant language, switching tended to elicit costs in RTs only if the nondominant language block was completed first, and elicited benefits in RTs if the dominant block was completed first; the same pattern was observed for the nondominant language, suggesting an order effect.

For error rates, bilinguals made fewer errors in the dominant language than in the nondominant language, a typical language dominance effect (*M =* 0.94% versus 2.54%; 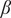 = 1.24; 95% CI = [0.70, 1.77], 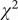 (1) = 20.56, *p* < .001). Other than that, error rate analyses did not reveal any significant results (*ps >* .16).

### 3.3. Discussion

In Experiment 2, we replicated the critical results in Peeters et al. (2014) and Li and Gollan (2022) – language switching was costly from semantic judgment to picture naming in the dominant language, particularly among more unbalanced bilinguals, supporting a shared inhibitory control mechanism between listening comprehension and production. Without salient orthographic cues, Chinese–English bilinguals strongly inhibited the dominant language throughout a testing block. Language switching from listening comprehension to production elicited benefits in the nondominant language, as in Li and Gollan (2022) and in our Experiment 1. Nevertheless, in Experiment 2, switch benefits were exclusively driven by those who completed the nondominant block first and were not modulated by the degree of bilingualism. Therefore, switch benefits in Experiment 2 may not be a result of language control. We will discuss the switch benefits in more detail in General Discussion.

## 4. Experiment 3: Listening comprehension and mixed-language picture naming

In Experiment 3, we examined whether language control within production may modulate or override language control between comprehension and production when salient orthographic cues were unavailable.

### 4.1. Method

#### 4.1.1. Participants

Thirty-two unbalanced Chinese–English bilingual undergraduates who did not participate in Experiments 1 and 2 were recruited from the same subject pool and participated for course credit (14 males and 18 females). Two subjects were removed because of too many invalid production trials (>20% were incorrect, triggered by noise, or no response within 4000 ms). Table 1 shows self-reported participant characteristics and MINT Sprint scores of the remaining participants. Twenty-four remaining participants were Chinese-dominant, and the other six were English-dominant. Participants from Experiments 2 and 3 did not differ in either self-reported or objectively measured proficiency levels of the two languages or any other variables (*ps >* .05; see Table 1).

#### 4.1.2. Design, materials and procedure

The design, materials and procedure were the same as those in Experiment 2, except that (1) there was only one block (with a break in the middle) for each subject, in which half of the pictures were named in English and the other half were named in Chinese, and (2) participants named each picture in either English or Chinese according to the national flag (either a US or a Chinese flag) above it.

### 4.2. Results

The procedures of data trimming and analyses were identical to those in Experiment 2, except that in Experiment 3, there was no block order variable, given that two picture-naming languages were mixed in the same block.

#### 4.2.1. Picture naming

For RTs, we removed 3.1% incorrect responses and another 4.2% of trials due to trimming procedures. Figure 1 (right panel) shows the mean RTs per language and per trial type. Unlike Experiments 1 and 2, participants showed a significant reversed dominance effect, producing the dominant language more slowly than the nondominant language (*M =* 2756 ms versus 2656 ms; 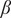 = 98 ms; 95% CI = [42 ms, 153 ms], 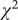 (1) = 18.74, *p* < .001). More critically, participants did not show an interaction between language and trial type or a trial type main effect (*ps >* .10). Following the previous two experiments, we still conducted analyses on each language separately.

For the dominant language, participants produced switch and nonswitch trials equally fast (*M =* 2753 ms versus 2759 ms; 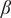 = − 7 ms; 95% CI = [−59 ms, 45 ms], 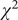 (1) < 1, *p =* .78). For the nondominant language, participants did not show a significant difference between switch and nonswitch trials either (*M =* 2635 ms versus 2677 ms; 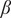 = − 48 ms; 95% CI = [−105 ms, 8 ms], 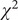 (1) = 2.87, *p =* .09). For both languages, additional analyses including BIS did not show significant interaction between BIS and trial type (*p*s > .53) or any BIS effects (*p*s > .20).^6^

We conducted a cross-experiment comparison between Experiments 2 and 3 on the dominant language as we did for Experiments 1 and 2, except that block order was not included as it did not exist in Experiment 3. Similarly, we showed a significant experiment * trial type interaction ( 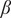 = 51; 95% CI = [4, 98], 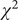 (1) = 4.44, *p =* .035).

Within Experiment 3, we conducted an exploratory analysis that focused on the influence of the preceding production trial language, omitting the intervening comprehension trial. This analysis aimed to address language control within production and revealed a significant interaction between trial type and language ( 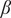 = − 71 ms; 95% CI = [−136 ms, −7 ms], 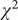 (1) = 4.68; *p = .*030). Further analyses showed significant language switch costs in the nondominant language (*M =* 2679 ms versus 2885 ms; 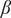 = 54 ms; 95% CI = [18 ms, 91 ms], 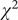 (1) = 8.43, *p =* .004), but not in the dominant language, despite a trend in the same direction (*M =* 2665 ms versus 2645 ms; 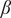 = 19 ms; 95% CI = [−66 ms, 29 ms], 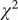 (1) < 1, *p =* .44). These results were likely affected by the intervening comprehension trials but provided suggestive evidence of within-production language control (i.e., switch costs within production).

The analysis of error rates focusing on language switching from comprehension to production showed two main effects. Bilinguals produced more errors in their dominant language than in the nondominant language, a reversed dominance effect that has been frequently found in production tasks (*M =* 4.37% versus 1.90%; 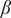 = − 1.01; 95% CI = [−1.65, −0.37], 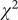 (1) *=* 9.53, *p =* .002). They produced fewer errors on switch than nonswitch trials (*M =* 2.57% versus 3.70%; 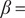 0.59; 95% CI = [0.03, 1.15], 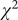 (1) = 4.22, *p =* .040). All other effects were nonsignificant (*ps >* .36). Table 2 shows the mean error rates in different languages and trial types.

#### 4.2.2. Semantic judgment

Table 5 shows RTs and error rates for semantic judgment responses in all conditions. In the RT analysis, we removed 2.1% incorrect responses and another 1.3% of trials due to trimming procedures. Neither RT nor error rate analyses revealed any significant results (*ps >* .05), but RTs in Experiment 3 were overall longer than those in Experiment 2 (*M =* 1476 ms versus 1296 ms; 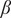 = 180 ms; 95% CI = [93 ms, 267 ms], 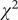 (1) = 16.45, *p* < .001).

**Table 5.** Mean response times and error rates (95% confidence intervals in brackets) for *semantic judgment* in Experiment 3 (listening comprehension + mixed-language picture naming)

Word language	Word trial type	RT (ms)	Error rate (%)
Dominant	Nonswitch	1475 [1455, 1494]	1.15 [0.61, 1.7]
	Switch	1450 [1432, 1469]	1.72 [1.06, 2.37]
Nondominant	Nonswitch	1488 [1466, 1510]	2.92 [2.06, 3.78]
	Switch	1486 [1467, 1505]	2.59 [1.79, 3.39]

*Note:* Overall, switch effects were small.

### 4.3. Discussion

In Experiment 3, we did not find language switch costs from comprehension to production in bilinguals’ dominant language, consistent with Liu et al. (2020, 2021). In addition, the BISs did not modulate switch effects in either language. These results suggested that Chinese–English bilinguals might not apply shared language control mechanisms across comprehension and production when both languages are used in both modalities. Further exploratory analysis showed switch costs within production. Although switch costs within production were only significant in the nondominant language, the trend in the dominant language followed the same pattern (i.e., switch costs). Considering that we did not manipulate the ratio of switch and nonswitch trials within production trials (currently, the ratio is about 0.9), better-controlled research is needed in the future to address this issue. Critically, the switch costs showed some suggestive evidence of language control within production, even with a comprehension trial between two production trials (note that in Liu et al., 2020, 2021, they showed switch costs on consecutive production trials), supporting our hypothesis that high language control demand within production may eliminate language switch costs between comprehension and production.

We also showed a reversed dominance effect in production trials, which might reflect proactive language control within the production task (see Goldrick & Gollan, 2023). That is, since bilinguals were aware that both languages would be produced within the same testing block, they proactively inhibited the dominant language to facilitate the production of the weaker nondominant language throughout the test.

## 5. General discussion

The present study examined whether language cues and control demand within production modulate bilinguals’ adaptive control of two languages between comprehension and production. In three experiments, Chinese–English bilinguals alternated between comprehension and production tasks on every trial. In the comprehension task, they judged the meaning of a written word (Experiment 1) or a spoken word (Experiment 2) in either language per testing block; in the production task, they named pictures in only one language (Experiments 1 and 2) or in either language (Experiment 3) per testing block. Although every trial involved task switching, only half involved language switching.

For picture naming in the dominant language, bilinguals exhibited significant language switch costs from comprehension to production in Experiment 2, particularly among more unbalanced bilinguals, but did not show any sign of language switch costs in Experiment 1 or Experiment 3. These contrasts suggested that both (a) providing salient orthographic cues (Experiment 1) and (b) involving both languages within production (Experiment 3) can eliminate language switch costs from comprehension to production in the dominant language. In other words, both factors can (or just one of them is enough to) avoid adopting shared language control mechanisms between comprehension and production. For the nondominant language, significant language switch benefits from comprehension to production were shown in Experiments 1 and 2. This effect was not significant in Experiment 3, but the numerical difference followed the same pattern. Lastly, when production was limited to one single language per testing block (Experiments 1 and 2), no language dominance effect appeared beyond a practice effect; when production involved both languages within a block (Experiment 3), a reversed dominance effect was shown, which might be a result of proactive inhibition on the dominant language within production.

### 5.1. The role of language cues

The comparison between Experiments 1 and 2 suggested that language cues played a critical role in inhibitory control between comprehension and production. In these two experiments, stimulus type (picture versus visual words; picture versus sound) informed language information, as pictures were always named in one language. However, comprehension trials included both languages. In Experiment 1, different from previous research using similar-script language pairs (e.g., Spanish–English), orthographic information served as an effective cue for rapid language identification by nature. Throughout a whole testing block, visual cues were effective in guiding language selection (Chinese scripts, English scripts and pictures that also refer to a single language within a block). In contrast, participants in our Experiment 2 might need to make more efforts to judge the language membership of words. The two experiments used the same language pairs, pictures and words, but the salient orthographic cues were no longer available in Experiment 2, and all words were produced by the same person (i.e., no interlocutor identity information either), so that participants had to use linguistic knowledge to judge each word (e.g., phonological and phonetic cues that are less immediately available and less perceptually salient than orthographic cues). This encouraged them to strongly inhibit the dominant language regardless of the task.

An alternative interpretation was that phonological information was not explicitly presented in comprehension trials in Experiment 1. However, even in Chinese, phonological information is automatically activated in visual word recognition (C. Li, Lin, et al., 2013a). In addition, Li and Gollan (2022) showed that even reading aloud that explicitly required phonological processing failed to elicit language switch costs in the following picture-naming trials. The absence of switch costs should not be due to other typological differences between Chinese and English either (e.g., differences in morphology or syntax), as the same language pairs and items were used in Experiments 1 and 2.

A third explanation was that bilinguals tended to strongly inhibit the dominant language under more demanding conditions (see discussion in Li & Gollan, 2022). Consistent with this idea, overall picture-naming latencies were longer in Experiment 2 than in Experiment 1, likely because the increased difficulty of recognizing spoken words added to the cognitive load during picture naming. However, naming latencies were even longer in Experiment 3, which nonetheless showed no language switch costs from comprehension to production. Therefore, while task difficulty clearly influenced overall RTs, it cannot account for cross-modality language switch cost patterns in the present study.

The orthographic cues account also most effectively explains cross-study differences among bilingual groups. French–English and Spanish–English bilinguals showed switch costs when alternating between semantic judgments of visual words and picture naming (Li & Gollan, 2022; Peeters et al., 2014), whereas the Chinese–English bilinguals in the present study did not, likely because orthographic cues are more salient between Chinese and English than in the other two language pairs. Extending the Adaptive Control Hypothesis across modalities, our results suggested that even when adopting the same experimental structure as previous research (bilingual mode in reading comprehension and monolingual mode in production), bilinguals still adaptively engage different control mechanisms depending on the effectiveness of available language cues.

Our findings revealed the transient reactive control mechanism between comprehension and production. This process is implemented when the nontarget language *disrupts* the selection of target language items, whereas another language control process – sustained proactive control – is implemented in *anticipation* of nontarget language interference (Declerck, 2020; Ma et al., 2016; Peeters & Dijkstra, 2018). While language cues appeared to affect the former process between comprehension and production, their influence on the latter process might not be robust. For example, several studies have shown that comprehending one language exclusively for a period of time could make subsequent picture naming in another language more difficult, a significant blocked language order effect across comprehension and production (Degani et al., 2020, 2024; Kreiner & Degani, 2015; Stasenko & Gollan, 2019). This sustained language immersion effect was robust among various types of language pairs, including Spanish–English, Hebrew–English, Russian–English and even American Sign Language–English, suggesting that participants proactively inhibited their dominant language for production when trying to comprehend the nondominant language. However, evidence for proactive control in comprehension is still limited (Declerck & Koch, 2023), and future research is needed to directly compare this influence on reactive versus proactive control within the same population in the same study.

### 5.2. Adaptive control according to the demands from the self-language system

The comparison between Experiments 2 and 3 suggested that language control between comprehension and production is affected by language control demands within production. Consistent with what Liu et al. (2020, 2021) hypothesized, when only one language was used throughout all production trials, nontarget language interference was minimal in the self-language system and language control within production was easy, so that language interference from others became salient and a shared language control mechanism between comprehension and production was adopted. When both languages were used within production, language selection within production was challenging and the nontarget language interference was mainly from the self-language system, so that separate language control mechanisms between comprehension and production were adopted. It might be true that the activation or inhibition of language nodes can be modulated by both endogenous and exogenous factors (the BIA-d model), which makes shared control mechanisms possible, but the weight of these factors differs. In production, exogenous factors seemed to play a secondary role and start to affect reactive control of two languages only when endogenous factors do not elicit strong nontarget language interference. These findings are consistent with the Adaptive Control Hypothesis and extend the scope of this hypothesis beyond pure production tasks – whether exogenous factors affect language control depends on the demands of language control within production.

Note that in the present study (as well as in Liu et al., 2020, 2021), both comprehension and production tasks focused on single-word processing, while Li and Gollan (2021) showed language switch costs from a comprehension-initiated task (reading aloud) to production in sentence contexts when both languages were involved in production. This is probably because sentence contexts bring another factor – default language selection. In sentence contexts, bilinguals often select a single language as the primary or default language in which most words are produced and that drives syntactic structure (Gollan & Goldrick, 2018; see the Matrix Language Framework model, Myers-Scotton & Jake, 2009). While switching out of the default language is error-prone, switching back to the default language appears to be easy and elicits very few language selection errors (Gollan & Goldrick, 2018). Therefore, the switch costs in Li and Gollan (2021) from reading aloud to picture naming might be a result of a violation of default language selection, which will be an interesting topic to address in future research. When the comprehension task involves sentence rather than single-word processing, subsequent production in another language may show language switch costs as a result of default language violation, regardless of how many potential target languages are involved in production.

Another contrast between Experiment 3 and the other two experiments was that only Experiment 3 showed the reversed dominance effect, a sign of proactive control. The previous two experiments did not show this effect, but the typical dominance effect was also missing – there was no significant difference between the two languages. Therefore, when production was limited to one language only, bilinguals might still proactively inhibit the dominant language, but to a lesser extent than when production might happen in both languages. Given that the practice effect (i.e., block order) might affect the language dominance effect in Experiments 1 and 2, future research is needed to better compare proactive control between comprehension and production in these two contexts (i.e., with versus without challenging language control within production).

### 5.3. Language switch benefits in the nondominant language

All three experiments in the present study showed language switch benefits or a trend of switch benefits from comprehension to production in the nondominant language, consistent with Li and Gollan (2022), which suggested that this result might reflect task switch costs between semantic judgment and naming. In the present study (and in Li & Gollan, 2022, Experiment 2), all trials involved task switching, and different task-set components (e.g., language, intention and response modality – manual versus vocal) may be integrated into a single task representation. This integration might make it easier to switch all components at once than keeping language constant but switching other components (see Philipp & Koch, 2010; Vandierendonck et al., 2008), leading to language switch benefits. Therefore, stronger switch benefits might reflect domain-general task switch effects. In our Experiment 2, for participants completing the nondominant block second, switch benefits to the nondominant language were eliminated, likely due to the familiarity of our paradigm. Such switch benefits did not show up in picture-naming trials in the dominant language, and Li and Gollan (2022) speculated that it was because the inhibition on the dominant language was so strong that language switch costs outweigh the task integration difficulty. However, this does not explain our results in Experiments 1 and 3, which showed no switch effects in the dominant language but consistent switch benefits in the nondominant language. While a speculation was that the task integration difficulty was more salient on more difficult trials (using the nondominant language is more difficult than using the dominant language), future research is needed to include task nonswitch trials to further explore what drives switch benefits. Despite this uncertainty, what is critical is that switch benefits appeared to be more about task switching than language switching. In contrast, switch costs in the dominant language in Experiment 2 were robust regardless of block order, not being modulated by task familiarity and reflecting more of a language effect.

### 5.4. Limitations and future directions

As mentioned above, future research may examine proactive control from comprehension to production, investigate the role of default language selection in language control across modalities and add task nonswitch trials (e.g., consecutive production or comprehension trials) to provide a more comprehensive view of cross-modality language switch. Adding task nonswitch trials can also address another limitation of this study – in Experiments 1 and 2, in each block, the ratio of two languages was 2:1 if considering all comprehension and production trials, as half of the comprehension trials shared the same language with the picture-naming language; in Experiment 3, the ratio of two languages was 1:1 for the entire block. This difference can be addressed by adding more comprehension trials and manipulating their languages well across experiments. However, this confound should not affect our critical results. With a 2:1 ratio, switching to the language used more often (i.e., the default language) in the block should be easier (Gollan & Goldrick, 2018). However, for the dominant language, we showed switch costs to the default language in Experiment 2 and did not show language switch costs when there was no default language in Experiment 3, suggesting that the default language of the entire block did not affect our critical results. Fourthly, the exploration of BIS effects may require a larger sample size in future research. Lastly, future research may explore other types of comprehension tasks. For example, judging whether a word contains a specific sound (Declerck & Philipp, 2018) more directly involves phonological processing and might elicit switch costs in subsequent production trials, even with salient cues and low control demands within production.

## 6. Conclusion

In summary, the present study suggested that whether bilinguals adopt a shared or separate language control mechanism when alternating between comprehension and production depends on multiple factors. Effective language cues may avoid the need to strongly inhibit the dominant language across comprehension and production. In addition, nontarget language interference in the self-language system might be more critical than languages produced by others, so that a shared language control mechanism across comprehension and production is less possible when language control within production is challenging. Together, these findings supported and extended the Adaptive Control Hypothesis, suggesting that bilinguals adaptively adopt different language control mechanisms across comprehension and production in different contexts.

## Data Availability

Data and R scripts of all three experiments are available on the Open Science Framework (https://osf.io/d3thn/?view_only=988ebf34364f4bdb811749a35123a603).

## Footnotes

1 Note that this does not apply to similar-script bilinguals such as Spanish–English bilinguals. When comprehension is unpredictable in bilinguals’ either language, participants need to rely on lexical knowledge rather than orthographic information to complete language selection.

2 The sample size was comparable with that in Li and Gollan (2022) and larger than that in Peeters et al. (2014). The present study closely followed the design of these two studies, and we used the same number of trials for each participant in them. In addition, power analysis based on language switch costs in Li and Gollan (2022, Experiment 2) using GPower (Erdfelder et al., 1996) also showed that 30 participants can reach 80% power threshold. Yet, following Brysbaert and Stevens (2018), future research with larger samples is desirable to further validate and extend the present findings with increased statistical power, as they recommend approximately 40 participants and 40 trials per condition for mixed-effects modeling.

3 An incorrect comprehension trial may indicate that participants did not process the word; it may fail to reveal its effect on the following picture trial.

4 We first included word category in the analysis, but the results showed that word category did not modulate the critical switch effects in any situation or any experiment. Therefore, we collapsed items after animal and nonanimal trials in all experiments.

5 We also conducted the same analysis in Experiment 1 and failed to show the interaction between BIS and trial type (*p =* .79) or any BIS effects (*ps >* .39). These results held when we only focused on more unbalanced bilinguals with a BIS ≤ 0.8 (i.e., more unbalanced bilinguals). In Experiment 1, these 18 participants still failed to show a trend of switch costs in the dominant language (*M* = 1684 ms vs. 1682 ms for nonswitch vs. switch trials*, p =* .99). In contrast, in Experiment 2, 26 participants with qualified BIS (≤0.8) still showed significant switch costs in the dominant language (*M* = 2151 ms vs. 2191 ms; *p* = .003).

6 Following the previous two experiments, when focusing on more unbalanced bilinguals with a BIS ≤ 0.8, the 25 qualified participants still failed to show a trend of language switch costs in the dominant language (*M* = 2769 ms vs. 2766 ms, *p* = .81).

## Appendix

### Pictures (English name) in Experiments 1–3

apple, backpack, box, bridge, candle, carrot, chair, strawberry, cloud, door, dress, egg, glass, hat, knife, leaf, meat, onion, orange, pencil, shoe, table, tree, umbrella, watch.

### Pictures (Chinese name) used in Experiments 1–3

苹果, 背包, 盒子, 桥, 蜡烛, 胡萝卜, 椅子, 草莓, 云, 门, 衣服, 鸡蛋, 杯子, 帽子, 刀, 叶子, 肉, 洋葱, 橙子, 铅笔, 鞋, 桌子, 树, 雨伞, 手表.

### English words in Experiments 1–3

bear, bird, cat, cow, dog, dolphin, duck, elephant, fox, frog, goat, penguin, monkey, mouse, owl, panda, pig, rabbit, kangaroo, sheep, squirrel, tiger, turtle, wolf, zebra, article, bread, butter, color, culture, event, flower, target, grass, group, joke, language, lettuce, library, meeting, movie, option, pain, science, soap, taste, tomato, trash, window, wine.

### Chinese words in Experiments 1–3

狗熊, 小鸟, 猫咪, 奶牛, 小狗, 海豚, 鸭子, 大象, 狐狸, 青蛙, 山羊, 企鹅, 猴子, 老鼠, 猫头鹰, 熊猫, 小猪, 兔子, 袋鼠, 绵羊, 松鼠, 老虎, 乌龟, 狼, 斑马, 文章, 面包, 黄油, 颜色, 文化, 事件, 鲜花, 目标, 青草, 团体, 玩笑, 语言, 生菜, 图书馆, 会议, 电影, 选项, 疼痛, 科学, 肥皂, 味道, 西红柿, 垃圾, 窗户, 葡萄酒.
